# Supplementary material for: Effectiveness of nirmatrelvir/ritonavir and molnupiravir in reducing the risk of short-term and long-term cardiovascular complications of COVID-19: a target trial emulation study
Source: Nat Commun. 2025 Dec 21;17:1031. doi: 10.1038/s41467-025-67776-4 (PMC12847700; doi:10.1038/s41467-025-67776-4)
Supplement: Supplementary file 1 — Supplementary Information [file 41467_2025_67776_MOESM1_ESM.pdf]

Supplementary Table 1. Target trial specification and emulation using observational data.

Protocol component	Target trial specification	Emulation using observational data
**Nirmatrelvir/ritonavir trial**		
Eligibility criteria	Aged over 18 years, with first-time confirmed SARS-CoV-2 infection diagnosis and hospital admission from March 11, 2022 to October 10, 2023.<br><br>Exclude patients who:<br>• Had a history of cardiovascular diseases on or before index date<br>• Died on index date<br>• Received nirmatrelvir/ritonavir or molnupiravir on or before index date<br>• Had drug contraindications to nirmatrelvir/ritonavir<br>• Had severe renal impairment (i.e., estimated glomerular filtration rate < 30 ml/min per 1·73 m^2, dialysis, or renal transplantation)<br>• Had severe liver impairment (i.e., cirrhosis, hepatocellular carcinoma, or liver transplantation)<br><br>The index date was defined as the date of SARS-CoV-2 diagnosis.	Same as for specification.<br><br>The date of confirmed SARS-CoV-2 infection diagnosis is the date of first positive RT-PCR during the enrolment period.<br><br>Patients were considered as COVID-19 hospitalizations if they were admitted to hospital within 3 days before or after the date of the confirmation of the SARS-CoV-2 infection.

Treatment strategies	Treatment arm: initiation of nirmatrelvir/ritonavir within 5 days of symptom onset according to the FDA-approved regimen. Standard of care followed in all other respects.<br>Control arm: standard of care followed in all other respects<br><br>Patients are expected to complete the full course of drugs.<br><br>Physicians can prescribe other concomitant drugs for patients in both treatment and control groups.<br><br>Patients in the treatment groups shall be censored if they receive an additional course of molnupiravir after nirmatrelvir/ritonavir treatment.	Same as for specification.<br><br>Date of positive RT-PCR is used as a proxy of date of symptom onset<br><br>It is assumed that once the patient initiated antiviral therapy, he/she will complete the full course of antiviral treatment.<br><br>Concomitant treatments at baseline are adjusted in the model.
Assignment procedures	Patients were assigned randomly to one of the treatment strategies and were informed of the assigned strategy.	Patients are classified into different treatment groups according to the prescription records within 5 days of index date.<br><br>Randomization of treatment assignments was emulated by cloning, censoring, and inverse probability of censoring weighting to address immortal time bias and post-assignment confounders

Outcomes	Incident cardiovascular complications occurred from 0-21 days and from 22-365 days following SARS-CoV-2 infection, including cardiovascular mortality, cerebrovascular disorders, dysrhythmia, ischemic heart disease, inflammatory heart disease, other cardiac disorders, thrombotic disorders, major adverse cardiac events, composite cardiovascular complications were determined based on ICD-9-CM and ICPC-2 code.<br><br>Outcomes were assessed from index date through day 21 and from 22-365.	Same as for specification.
Follow-up	For each person, follow-up started on the day of treatment randomization and continued until day 21 for short-term outcomes or from day 22-365 for long-term outcomes.	Same as for specification.<br><br>Patients were followed until the occurrence of outcome events, death, end of outcome assessment period, or the end of data availability, whichever came first.
Causal contrast	Per-protocol effect.<br>Intention-to-treat effect.	Observational analogue of per-protocol effect.
Identifying assumptions	Within levels of the adjustment of baseline confounders, groups receiving each treatment strategy at each time have the same counterfactual risk of the outcome (conditional exchangeability) and there is a non-zero probability of receiving each treatment strategy at each time.<br><br>The treatment strategies are sufficiently well defined.	Same as for specification.<br>Cloning, censoring and inverse probability of censoring weighting were used to address informative censoring and baseline confounders.
Data analysis plan	Measure the cause-specific hazard ratio of cardiovascular complications across treatment strategies.	Estimate the cause-specific hazard ratio of cardiovascular complications with inverse probability of censoring weighting considered and death as a competing risk by using the cause-specific Cox proportional hazard model.

Molnupiravir trial		
Eligibility criteria	Aged over 18 years, with first-time confirmed SARS-CoV-2 infection diagnosis and hospital admission from March 11, 2022 to October 10, 2023.<br><br>Exclude patients who:<br>• Had a history of cardiovascular diseases on or before index date<br>• Dead on index date<br>• Received nirmatrelvir/ritonavir or molnupiravir on or before index date<br>• Had drug contraindications to nirmatrelvir/ritonavir<br><br>The index date was defined as the date of SARS-CoV-2 diagnosis.	Same as for specification.<br><br>The date of confirmed SARS-CoV-2 infection diagnosis is the date of first positive RT-PCR during the enrolment period.<br><br>Patients were considered as COVID-19 hospitalizations if they were admitted to hospital within 3 days before or after the date of the confirmation of the SARS-CoV-2 infection.
Treatment strategies	Treatment arm: initiation of molnupiravir within 5 days of symptom onset according to the FDA-approved regimen. Standard of care followed in all other respects.<br>Control arm: standard of care followed in all other respects<br><br>Patients are expected to complete the full course of drugs.<br><br>Physicians can prescribe other concomitant drugs for patients in both treatment and control groups.	Same as for specification.<br><br>Date of positive RT-PCR is used as a proxy of date of symptom onset<br><br>It is assumed that once the patient initiated antiviral therapy, he/she will complete the full course of antiviral treatment.<br><br>Concomitant treatments at baseline are adjusted in the model.

	Patients in the treatment groups shall be censored if they receive an additional course of nirmatrelvir/ritonavir after molnupiravir treatment.	
Assignment procedures	Patients were assigned randomly to one of the treatment strategies and were informed of the assigned strategy.	Patients are classified into different treatment groups according to the prescription records within 5 days of index date.<br><br>Randomization of treatment assignments was emulated by cloning, censoring, and inverse probability of censoring weighting to address immortal time bias and post-assignment confounders
Outcomes	Incident cardiovascular complications occurred from 0-21 days and from 22-365 days following SARS-CoV-2 infection, including cardiovascular mortality, cerebrovascular disorders, dysrhythmia, ischemic heart disease, inflammatory heart disease, other cardiac disorders, thrombotic disorders, major adverse cardiac events, composite cardiovascular complications were determined based on ICD-9-CM and ICPC-2 code.<br><br>Outcomes were assessed from index date through day 21 and from 22-365.	Same as for specification.
Follow-up	For each person, follow-up started on the day of treatment randomization and continued until day 21 for short-term outcomes or from day 22-365 for long-term outcomes.	Same as for specification.<br><br>Patients were followed until the occurrence of outcome events, death, end of outcome assessment period, or the end of data availability, whichever came first.
Causal contrast	Per-protocol effect.<br>Intention-to-treat effect.	Observational analogue of per-protocol effect.

Identifying assumptions	Within levels of the adjustment of baseline confounders, groups receiving each treatment strategy at each time have the same counterfactual risk of the outcome (conditional exchangeability) and there is a non-zero probability of receiving each treatment strategy at each time.<br><br>The treatment strategies are sufficiently well defined.	Same as for specification.<br>Cloning, censoring and inverse probability of censoring weighting were used to address informative censoring and baseline confounders.
Data analysis plan	Measure the cause-specific hazard ratio of cardiovascular complications across treatment strategies.	Estimate the cause-specific hazard ratio of cardiovascular complications with inverse probability of censoring weighting considered and death as a competing risk by using the cause-specific Cox proportional hazard model.

Supplementary Table 2. Codes used to define cardiovascular outcomes.

Disease	Code (ICD-9 or ICPC-2)
Cerebrovascular disorders	433, 434, 435, 436, K89, K90
Dysrhythmia	427.0, 427.3, 427.4, 427.6, 427.8, 427.9, 785.0, K05, K78, K79
Ischemic heart disease	410, 411, 413, 414.8, K74, K75, K76
Inflammatory heart disease	393, 398.0, 420, 422, 423, 429.0
Other cardiac disorders	425.4, 427.5, 428, 785.51, K77
Thrombotic disorders	415, 451.1, 451.8, 451.9, 453, K93, K94
Major adverse cardiac events	410, 427.4, 427.5, 427.6, 427.8, 427.9, 428, 430, 431, 432, 433, 434, 436, K75, K77, K90
Composite cardiovascular complications	First incident of any cardiovascular outcomes investigated in this study
Cardiovascular mortality	Deceased within 30 days after diagnosis of any cardiovascular diseases investigated in this study

Supplementary Table 3. Code used to define ventilatory support.

Procedure	Code (ICD-9)
Extracorporeal membrane oxygenation (ECMO)	39.65
Other sleep disorder function tests	89.18
Non-invasive mechanical ventilation	93.9
Hyperbaric oxygenation	93.95
Other oxygen enrichment	93.96
Other continuous invasive mechanical ventilation	96.7x
Insertion of endotracheal tube	96.04

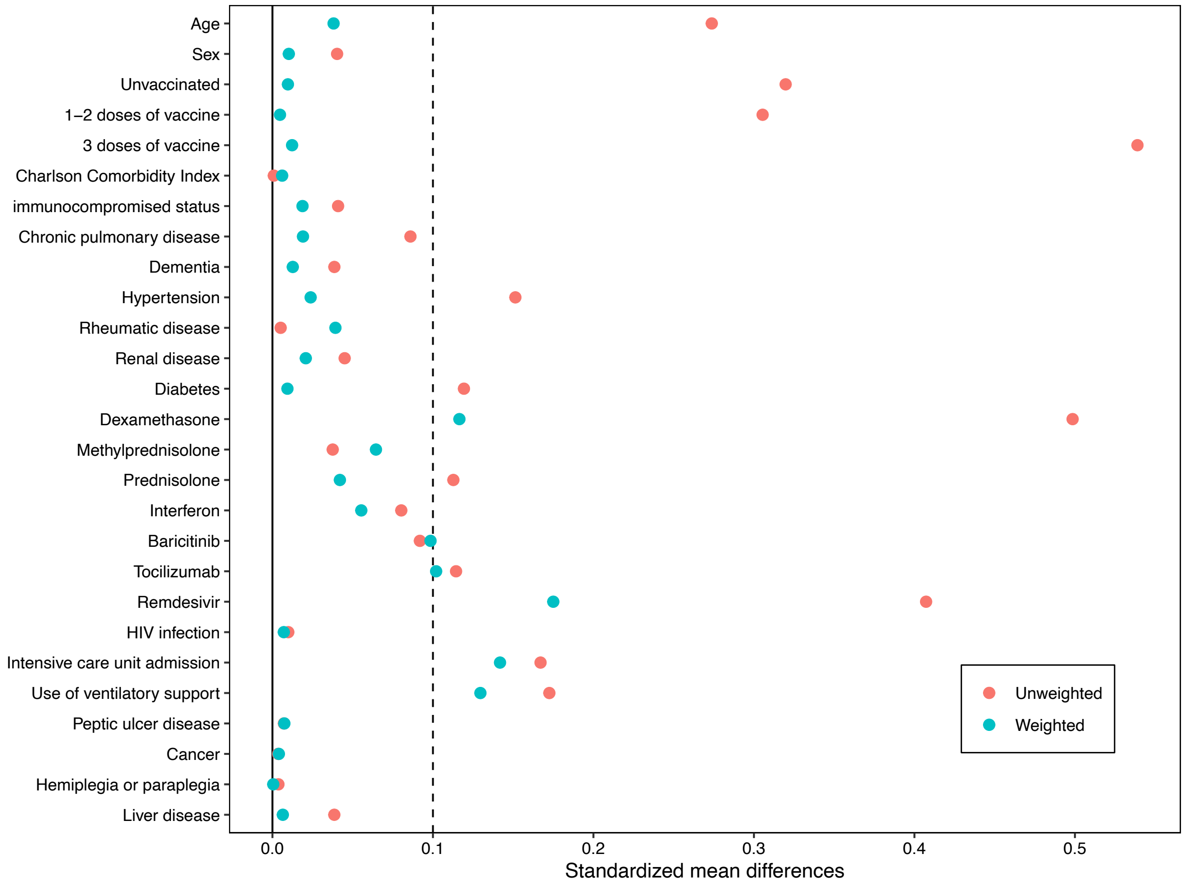

Supplementary Fig. 1. Absolute standardized mean differences of baseline covariates before (red dots) and after weighting (blue dots) between nirmatrelvir/ritonavir recipients (n=14,842) and controls (n=19,660) in nirmatrelvir/ritonavir trial. The solid line marks an SMD of 0.0 and the dash line marks an absolute SMD of 0.1.

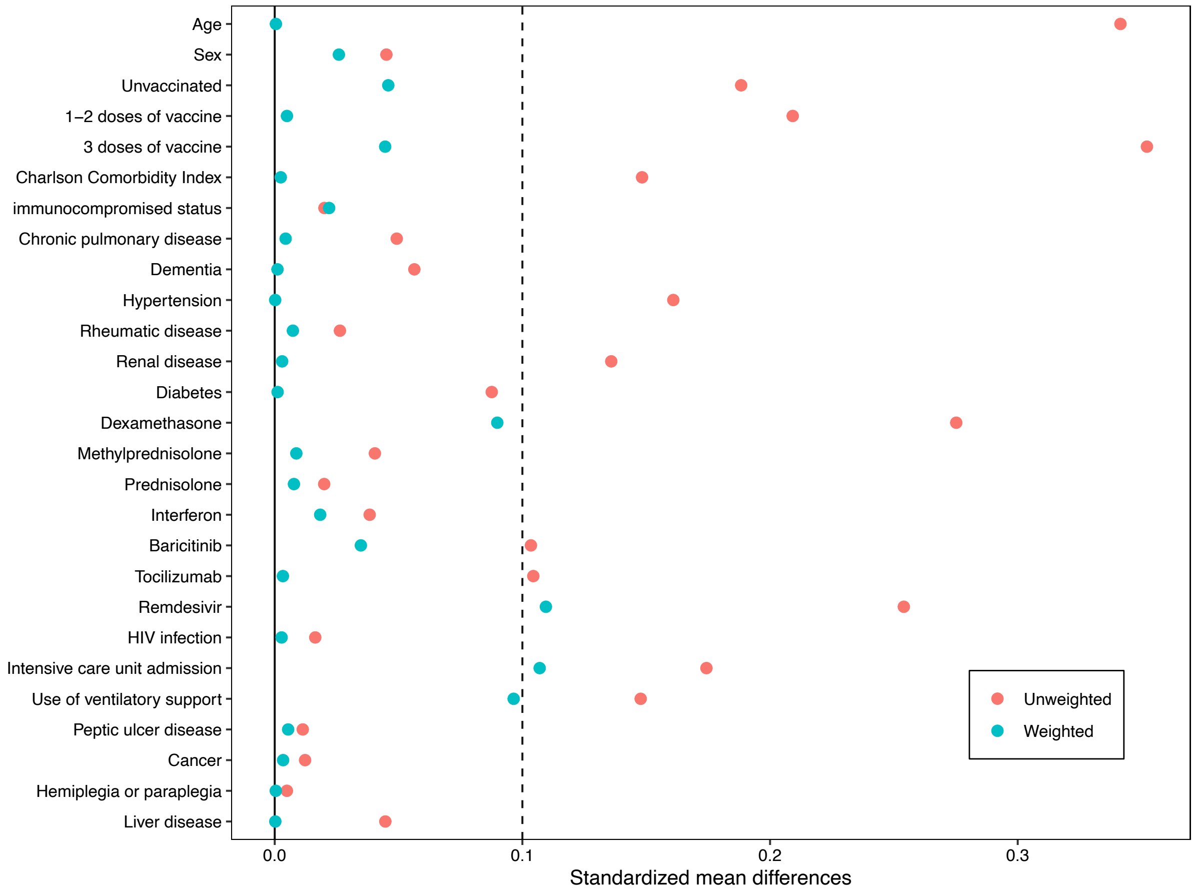

Supplementary Fig. 2. Absolute standardized mean differences of baseline covariates before (red dots) and after weighting (blue dots) between molnupiravir recipients (n=10,053) and controls (n=22,163) in molnupiravir trial. The solid line marks an SMD of 0.0 and the dash line marks an absolute SMD of 0.1.

(A) Nirmatrelvir/ritonavir trial

Outcome	Outcome assessment period	Adjusted RD (95% CI)	Adjusted HR (95% CI)	P value
Cardiovascular mortality				
	day 0–21	−1.19% (−1.53 to −0.84)	0.53 (0.32 to 0.90)	0.018
	day 22–365	−0.98% (−1.51 to −0.45)	0.69 (0.52 to 0.92)	0.011
Composite cardiovascular complications				
	day 0–21	−3.53% (−4.38 to −2.69)	0.57 (0.47 to 0.70)	<0.001
	day 22–365	−1.59% (−2.47 to −0.71)	0.80 (0.68 to 0.95)	0.009
MACE				
	day 0–21	−2.45% (−3.07 to −1.84)	0.56 (0.42 to 0.76)	0.000
	day 22–365	−1.36% (−2.10 to −0.61)	0.77 (0.62 to 0.95)	0.016
Cerebrovascular disorders				
	day 0–21	−1.18% (−1.63 to −0.73)	0.37 (0.26 to 0.52)	<0.001
	day 22–365	−0.76% (−1.20 to −0.32)	0.62 (0.49 to 0.79)	<0.001
Dysrhythmia				
	day 0–21	−0.82% (−1.31 to −0.33)	0.69 (0.55 to 0.87)	0.001
	day 22–365	−0.67% (−1.17 to −0.16)	0.72 (0.59 to 0.88)	0.001
Ischemic heart disease				
	day 0–21	−1.15% (−1.58 to −0.72)	0.65 (0.39 to 1.07)	0.091
	day 22–365	−0.22% (−0.64 to 0.20)	0.82 (0.63 to 1.05)	0.118
Inflammatory heart disease				
	day 0–21	−0.05% (−0.27 to 0.16)	0.98 (0.13 to 7.53)	0.981
	day 22–365	−0.08% (−0.16 to −0.01)	0.36 (0.14 to 0.94)	0.036
Other cardiac disorders				
	day 0–21	−1.02% (−1.35 to −0.68)	0.57 (0.32 to 1.00)	0.050
	day 22–365	−0.87% (−1.46 to −0.29)	0.84 (0.62 to 1.14)	0.253
Thrombotic disorders				
	day 0–21	−0.15% (−0.30 to −0.01)	0.50 (0.25 to 0.99)	0.047
	day 22–365	−0.15% (−0.39 to 0.09)	0.76 (0.45 to 1.29)	0.313

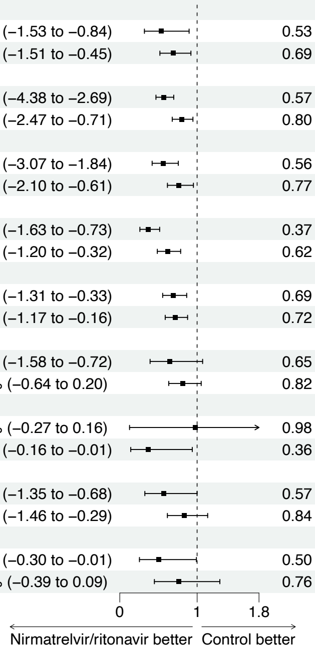

(B) Molnupiravir trial

Outcome	Outcome assessment period	Adjusted RD (95% CI)	Adjusted HR (95% CI)	P value
Cardiovascular mortality				
	day 0–21	−0.88% (−1.38 to −0.38)	0.68 (0.54 to 0.85)	0.001
	day 22–365	−0.29% (−0.87 to 0.28)	0.89 (0.74 to 1.07)	0.198
Composite cardiovascular complications				
	day 0–21	−1.48% (−2.36 to −0.60)	0.79 (0.70 to 0.89)	<0.001
	day 22–365	−0.59% (−1.51 to 0.33)	0.90 (0.80 to 1.00)	0.060
MACE				
	day 0–21	−1.11% (−1.75 to −0.47)	0.74 (0.63 to 0.87)	<0.001
	day 22–365	−0.49% (−1.27 to 0.30)	0.88 (0.77 to 1.02)	0.084
Cerebrovascular disorders				
	day 0–21	−1.01% (−1.45 to −0.56)	0.48 (0.35 to 0.67)	<0.001
	day 22–365	−0.41% (−0.88 to 0.06)	0.79 (0.62 to 0.99)	0.043
Dysrhythmia				
	day 0–21	−0.22% (−0.77 to 0.33)	0.89 (0.74 to 1.08)	0.253
	day 22–365	−0.01% (−0.57 to 0.55)	0.97 (0.81 to 1.15)	0.702
Ischemic heart disease				
	day 0–21	−0.03% (−0.48 to 0.42)	0.95 (0.77 to 1.17)	0.621
	day 22–365	−0.06% (−0.54 to 0.41)	0.94 (0.75 to 1.18)	0.576
Inflammatory heart disease				
	day 0–21	−0.03% (−0.21 to 0.16)	0.72 (0.09 to 6.03)	0.762
	day 22–365	−0.01% (−0.11 to 0.09)	0.88 (0.37 to 2.11)	0.781
Other cardiac disorders				
	day 0–21	−0.71% (−1.15 to −0.26)	0.68 (0.54 to 0.86)	0.002
	day 22–365	−0.02% (−0.66 to 0.62)	0.96 (0.81 to 1.14)	0.641
Thrombotic disorders				
	day 0–21	−0.22% (−0.37 to −0.06)	0.45 (0.25 to 0.80)	0.007
	day 22–365	−0.11% (−0.36 to 0.15)	0.83 (0.56 to 1.23)	0.355

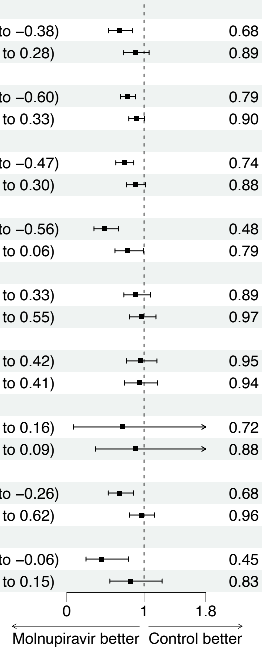

Supplementary Fig. 3. Risk of cardiovascular complications in target trials among hospitalized patients aged > 65 years with COVID-19. (A) Target trial of nirmatrelvir/ritonavir (n=11,212) versus no treatment (n=12,296). (B) Target trial of molnupiravir (n=7,781) versus no treatment (n=14,317). Adjusted HRs (square dots) and 95% CIs (error bars) are presented in (A) and (B). The dashed vertical line in (A) and (B) represents the HR of 1.00. Statistical analysis with two-sided Wald test in (A) and (B). MACE: major adverse cardiovascular events. RD: risk difference. HR: hazard ratio. CI: confidence interval.

(A) Nirmatrelvir/ritonavir trial

Outcome	Outcome assessment period	Adjusted RD (95% CI)	Adjusted HR (95% CI)	P value
Cardiovascular mortality				
	day 0–21	−0.31% (−0.52 to −0.11)	0.55 (0.31 to 0.98)	0.043
	day 22–365	−0.12% (−0.42 to 0.18)	0.91 (0.32 to 2.57)	0.862
Composite cardiovascular complications				
	day 0–21	−1.74% (−2.47 to −1.01)	0.60 (0.46 to 0.79)	<0.001
	day 22–365	−0.47% (−1.09 to 0.15)	0.73 (0.50 to 1.09)	0.124
MACE				
	day 0–21	−1.14% (−1.62 to −0.66)	0.48 (0.33 to 0.71)	<0.001
	day 22–365	−0.25% (−0.61 to 0.11)	0.79 (0.44 to 1.42)	0.428
Cerebrovascular disorders				
	day 0–21	−0.55% (−0.86 to −0.24)	0.48 (0.26 to 0.89)	0.019
	day 22–365	0.01% (−0.13 to 0.14)	1.22 (0.56 to 2.69)	0.613
Dysrhythmia				
	day 0–21	0.02% (−0.43 to 0.47)	1.04 (0.68 to 1.60)	0.843
	day 22–365	−0.25% (−0.53 to 0.02)	0.49 (0.23 to 1.05)	0.065
Ischemic heart disease				
	day 0–21	−0.91% (−1.23 to −0.60)	0.10 (0.03 to 0.30)	<0.001
	day 22–365	−0.26% (−0.53 to 0.02)	0.50 (0.24 to 1.04)	0.063
Inflammatory heart disease				
	day 0–21	0.08% (0.01 to 0.15)	1.55 (0.38 to 6.35)	0.541
	day 22–365	0.01% (−0.08 to 0.09)	0.69 (0.16 to 3.02)	0.618
Other cardiac disorders				
	day 0–21	−0.54% (−0.81 to −0.27)	0.50 (0.27 to 0.93)	0.029
	day 22–365	−0.16% (−0.48 to 0.16)	0.52 (0.25 to 1.07)	0.076
Thrombotic disorders				
	day 0–21	−0.05% (−0.23 to 0.13)	1.50 (0.80 to 2.81)	0.208
	day 22–365	−0.07% (−0.29 to 0.14)	0.67 (0.31 to 1.46)	0.317

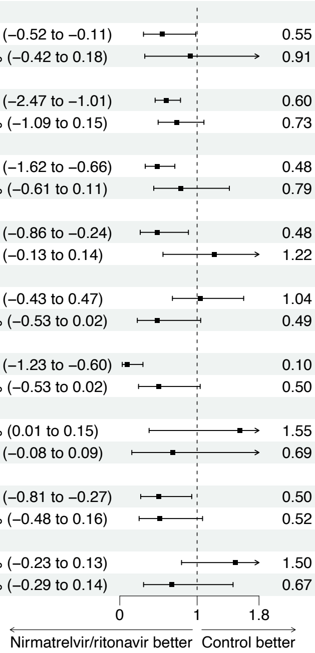

(B) Molnupiravir trial

Outcome	Outcome assessment period	Adjusted RD (95% CI)	Adjusted HR (95% CI)	P value
Cardiovascular mortality				
	day 0–21	−0.08% (−0.27 to 0.11)	0.49 (0.22 to 1.08)	0.077
	day 22–365	−0.22% (−0.42 to −0.03)	0.43 (0.19 to 0.95)	0.037
Composite cardiovascular complications				
	day 0–21	−0.22% (−1.22 to 0.78)	0.88 (0.65 to 1.19)	0.411
	day 22–365	0.63% (−0.22 to 1.48)	1.09 (0.79 to 1.50)	0.601
MACE				
	day 0–21	−0.18% (−0.99 to 0.64)	0.85 (0.59 to 1.24)	0.408
	day 22–365	0.31% (−0.25 to 0.87)	1.20 (0.80 to 1.81)	0.380
Cerebrovascular disorders				
	day 0–21	−0.35% (−0.77 to 0.06)	0.76 (0.45 to 1.28)	0.305
	day 22–365	0.19% (−0.18 to 0.55)	1.29 (0.75 to 2.21)	0.351
Dysrhythmia				
	day 0–21	0.34% (−0.19 to 0.86)	1.37 (0.81 to 2.33)	0.240
	day 22–365	0.29% (−0.19 to 0.76)	1.27 (0.68 to 2.35)	0.458
Ischemic heart disease				
	day 0–21	0.11% (−0.41 to 0.62)	1.07 (0.62 to 1.85)	0.807
	day 22–365	0.11% (−0.34 to 0.56)	1.00 (0.53 to 1.88)	0.998
Inflammatory heart disease				
	day 0–21	0.02% (−0.08 to 0.13)	1.61 (0.29 to 8.89)	0.585
	day 22–365	0.03% (−0.11 to 0.16)	0.66 (0.10 to 4.24)	0.665
Other cardiac disorders				
	day 0–21	−0.18% (−0.57 to 0.21)	0.61 (0.31 to 1.21)	0.154
	day 22–365	−0.05% (−0.42 to 0.31)	0.74 (0.42 to 1.32)	0.310
Thrombotic disorders				
	day 0–21	−0.06% (−0.29 to 0.17)	0.75 (0.21 to 2.77)	0.672
	day 22–365	0.08% (−0.25 to 0.41)	0.97 (0.43 to 2.19)	0.936

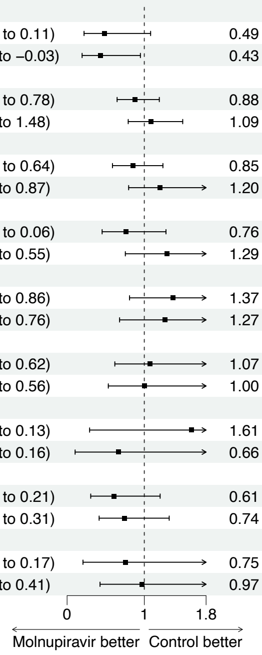

Supplementary Fig. 4. Risk of cardiovascular complications in target trials among hospitalized patients aged ≤ 65 years with COVID-19. (A) Target trial of nirmatrelvir/ritonavir (n=3,630) versus no treatment (n=7,364). (B) Target trial of molnupiravir versus (n=2,272) no treatment (n=7,846). Adjusted HRs (square dots) and 95% CIs (error bars) are presented in (A) and (B). The dashed vertical line in (A) and (B) represents the HR of 1.00. Statistical analysis with two-sided Wald test in (A) and (B). MACE: major adverse cardiovascular events. RD: risk difference. HR: hazard ratio. CI: confidence interval.

(A) Nirmatrelvir/ritonavir trial

Outcome	Outcome assessment period	Adjusted RD (95% CI)	Adjusted HR (95% CI)	P value
Cardiovascular mortality				
	day 0–21	−1.24% (−1.69 to −0.78)	0.27 (0.17 to 0.44)	<0.001
	day 22–365	−0.47% (−0.93 to −0.01)	0.58 (0.40 to 0.83)	0.003
Composite cardiovascular complications				
	day 0–21	−2.76% (−3.54 to −1.99)	0.53 (0.43 to 0.64)	<0.001
	day 22–365	−1.42% (−2.20 to −0.64)	0.74 (0.57 to 0.97)	0.026
MACE				
	day 0–21	−1.89% (−2.46 to −1.32)	0.50 (0.38 to 0.66)	<0.001
	day 22–365	−1.19% (−1.86 to −0.52)	0.78 (0.56 to 1.08)	0.139
Cerebrovascular disorders				
	day 0–21	−0.88% (−1.34 to −0.43)	0.45 (0.27 to 0.75)	0.002
	day 22–365	−0.10% (−0.53 to 0.32)	0.57 (0.37 to 0.89)	0.013
Dysrhythmia				
	day 0–21	−0.79% (−1.26 to −0.32)	0.61 (0.46 to 0.80)	<0.001
	day 22–365	−0.78% (−1.30 to −0.25)	0.58 (0.44 to 0.76)	<0.001
Ischemic heart disease				
	day 0–21	−0.80% (−1.16 to −0.44)	0.40 (0.27 to 0.59)	<0.001
	day 22–365	−0.02% (−0.34 to 0.31)	0.74 (0.53 to 1.03)	0.078
Inflammatory heart disease				
	day 0–21	−0.01% (−0.16 to 0.13)	1.31 (0.16 to 11.04)	0.802
	day 22–365	−0.01% (−0.10 to 0.07)	0.58 (0.18 to 1.89)	0.365
Other cardiac disorders				
	day 0–21	−0.89% (−1.22 to −0.56)	0.31 (0.19 to 0.51)	<0.001
	day 22–365	−0.70% (−1.21 to −0.19)	0.85 (0.58 to 1.26)	0.427
Thrombotic disorders				
	day 0–21	−0.05% (−0.19 to 0.08)	0.98 (0.46 to 2.12)	0.961
	day 22–365	0.08% (−0.15 to 0.31)	0.84 (0.49 to 1.43)	0.519

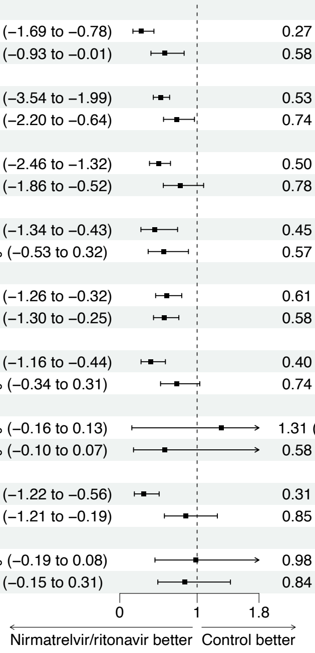

(B) Molnupiravir trial

Outcome	Outcome assessment period	Adjusted RD (95% CI)	Adjusted HR (95% CI)	P value
Cardiovascular mortality				
	day 0–21	−1.25% (−1.74 to −0.76)	0.52 (0.38 to 0.71)	<0.001
	day 22–365	−0.37% (−0.91 to 0.17)	0.83 (0.64 to 1.06)	0.138
Composite cardiovascular complications				
	day 0–21	−0.84% (−1.77 to 0.09)	0.81 (0.68 to 0.96)	0.014
	day 22–365	0.35% (−0.62 to 1.32)	0.99 (0.85 to 1.15)	0.866
MACE				
	day 0–21	−0.88% (−1.58 to −0.18)	0.70 (0.57 to 0.87)	0.001
	day 22–365	0.17% (−0.65 to 1.00)	0.98 (0.81 to 1.18)	0.842
Cerebrovascular disorders				
	day 0–21	−0.69% (−1.08 to −0.30)	0.47 (0.32 to 0.70)	<0.001
	day 22–365	−0.08% (−0.56 to 0.41)	0.90 (0.66 to 1.24)	0.524
Dysrhythmia				
	day 0–21	−0.05% (−0.63 to 0.53)	0.93 (0.73 to 1.19)	0.553
	day 22–365	0.00% (−0.63 to 0.63)	0.95 (0.75 to 1.21)	0.681
Ischemic heart disease				
	day 0–21	0.31% (−0.19 to 0.81)	1.02 (0.77 to 1.35)	0.884
	day 22–365	−0.09% (−0.51 to 0.32)	0.88 (0.64 to 1.21)	0.430
Inflammatory heart disease				
	day 0–21	−0.04% (−0.13 to 0.05)	0.79 (0.15 to 4.30)	0.786
	day 22–365	−0.02% (−0.12 to 0.08)	0.62 (0.12 to 3.19)	0.572
Other cardiac disorders				
	day 0–21	−0.63% (−1.05 to −0.22)	0.62 (0.44 to 0.89)	0.010
	day 22–365	0.30% (−0.35 to 0.95)	1.06 (0.84 to 1.33)	0.606
Thrombotic disorders				
	day 0–21	0.01% (−0.06 to 0.05)	0.93 (0.43 to 2.01)	0.851
	day 22–365	0.12% (−0.18 to 0.43)	1.09 (0.69 to 1.72)	0.715

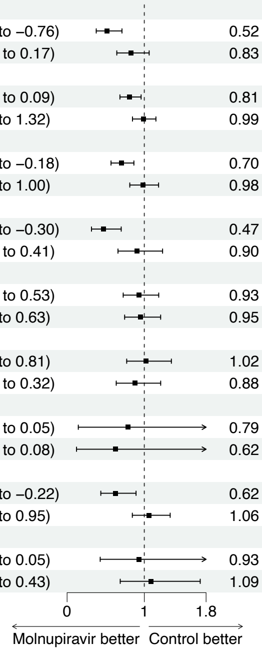

Supplementary Fig. 5. Risk of cardiovascular complications in target trials among female hospitalized patients with COVID-19. (A) Target trial of nirmatrelvir/ritonavir (n=7,344) versus no treatment (n=10,124). (B) Target trial of molnupiravir (n=4,894) versus no treatment (n=11,289). Adjusted HRs (square dots) and 95% CIs (error bars) are presented in (A) and (B). The dashed vertical line in (A) and (B) represents the HR of 1.00. Statistical analysis with two-sided Wald test in (A) and (B).

MACE: major adverse cardiovascular events. RD: risk difference. HR: hazard ratio. CI: confidence interval.

(A) Nirmatrelvir/ritonavir trial

Outcome	Outcome assessment period	Adjusted RD (95% CI)	Adjusted HR (95% CI)	P value
Cardiovascular mortality				
	day 0–21	−0.79% (−1.08 to −0.50)	0.61 (0.42 to 0.89)	0.009
	day 22–365	−0.89% (−1.46 to −0.32)	0.67 (0.45 to 1.00)	0.049
Composite cardiovascular complications				
	day 0–21	−3.43% (−4.37 to −2.48)	0.56 (0.46 to 0.68)	<0.001
	day 22–365	−1.66% (−2.64 to −0.68)	0.74 (0.62 to 0.89)	0.001
MACE				
	day 0–21	−2.43% (−3.13 to −1.73)	0.46 (0.34 to 0.61)	<0.001
	day 22–365	−1.62% (−2.42 to −0.81)	0.63 (0.51 to 0.77)	<0.001
Cerebrovascular disorders				
	day 0–21	−0.99% (−1.52 to −0.46)	0.42 (0.27 to 0.66)	<0.001
	day 22–365	−0.43% (−0.94 to 0.08)	0.72 (0.53 to 0.98)	0.037
Dysrhythmia				
	day 0–21	−0.50% (−1.03 to 0.02)	0.76 (0.58 to 1.00)	0.046
	day 22–365	−0.58% (−1.11 to −0.06)	0.70 (0.53 to 0.91)	0.009
Ischemic heart disease				
	day 0–21	−1.50% (−2.03 to −0.98)	0.37 (0.22 to 0.62)	<0.001
	day 22–365	−0.28% (−0.75 to 0.18)	0.79 (0.58 to 1.08)	0.144
Inflammatory heart disease				
	day 0–21	0.01% (−0.05 to 0.07)	1.23 (0.24 to 6.36)	0.806
	day 22–365	−0.08% (−0.17 to 0.01)	0.39 (0.13 to 1.20)	0.100
Other cardiac disorders				
	day 0–21	−0.96% (−1.33 to −0.59)	0.62 (0.43 to 0.89)	0.010
	day 22–365	−1.23% (−1.90 to −0.55)	0.61 (0.42 to 0.88)	0.009
Thrombotic disorders				
	day 0–21	−0.20% (−0.37 to −0.02)	1.33 (0.65 to 2.72)	0.429
	day 22–365	−0.25% (−0.50 to 0.01)	0.52 (0.30 to 0.89)	0.017

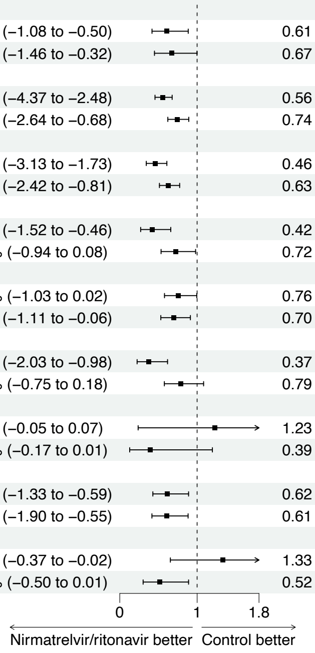

(B) Molnupiravir trial

Outcome	Outcome assessment period	Adjusted RD (95% CI)	Adjusted HR (95% CI)	P value
Cardiovascular mortality				
	day 0–21	−0.73% (−1.21 to −0.26)	0.76 (0.56 to 1.01)	0.062
	day 22–365	−0.42% (−1.03 to 0.19)	0.80 (0.63 to 1.03)	0.079
Composite cardiovascular complications				
	day 0–21	−1.66% (−2.67 to −0.66)	0.76 (0.66 to 0.89)	0.001
	day 22–365	−0.99% (−2.03 to 0.04)	0.84 (0.72 to 0.97)	0.018
MACE				
	day 0–21	−1.01% (−1.76 to −0.25)	0.76 (0.63 to 0.93)	0.007
	day 22–365	−0.72% (−1.60 to 0.15)	0.83 (0.69 to 1.00)	0.052
Cerebrovascular disorders				
	day 0–21	−0.63% (−1.17 to −0.08)	0.63 (0.44 to 0.92)	0.017
	day 22–365	−0.33% (−0.90 to 0.24)	0.81 (0.60 to 1.10)	0.181
Dysrhythmia				
	day 0–21	−0.10% (−0.69 to 0.48)	0.93 (0.72 to 1.19)	0.546
	day 22–365	−0.24% (−0.80 to 0.32)	0.86 (0.68 to 1.10)	0.238
Ischemic heart disease				
	day 0–21	−0.31% (−0.86 to 0.24)	0.84 (0.64 to 1.10)	0.212
	day 22–365	0.17% (−0.38 to 0.73)	1.03 (0.78 to 1.35)	0.833
Inflammatory heart disease				
	day 0–21	−0.01% (−0.08 to 0.05)	1.03 (0.20 to 5.19)	0.971
	day 22–365	0.05% (−0.09 to 0.19)	1.34 (0.54 to 3.33)	0.524
Other cardiac disorders				
	day 0–21	−0.60% (−1.11 to −0.09)	0.74 (0.55 to 1.01)	0.057
	day 22–365	−0.61% (−1.31 to 0.10)	0.79 (0.62 to 1.00)	0.050
Thrombotic disorders				
	day 0–21	−0.33% (−0.50 to −0.16)	0.32 (0.14 to 0.73)	0.006
	day 22–365	−0.36% (−0.61 to −0.12)	0.43 (0.24 to 0.78)	0.005

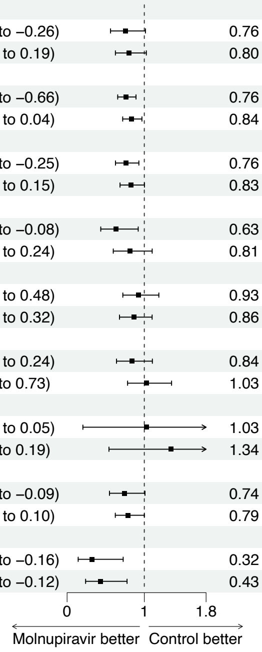

Supplementary Fig. 6. Risk of cardiovascular complications in target trials among male hospitalized patients with COVID-19. (A) Target trial of nirmatrelvir/ritonavir (n=7,498) versus no treatment (n=9,536). (B) Target trial of molnupiravir (n=5,159) versus no treatment (n=10,874). Adjusted HRs (square dots) and 95% CIs (error bars) are presented in (A) and (B). The dashed vertical line in (A) and (B) represents the HR of 1.00. Statistical analysis with two-sided Wald test in (A) and (B).

MACE: major adverse cardiovascular events. RD: risk difference. HR: hazard ratio. CI: confidence interval.

(A) Nirmatrelvir/ritonavir trial

Outcome	Outcome assessment period	Adjusted RD (95% CI)	Adjusted HR (95% CI)	P value
Cardiovascular mortality				
	day 0–21	−1.03% (−1.66 to −0.39)	0.43 (0.24 to 0.79)	0.007
	day 22–365	−0.43% (−1.48 to 0.62)	0.83 (0.53 to 1.29)	0.405
Composite cardiovascular complications				
	day 0–21	−2.84% (−4.09 to −1.60)	0.56 (0.41 to 0.75)	<0.001
	day 22–365	−2.37% (−4.14 to −0.61)	0.74 (0.57 to 0.96)	0.022
MACE				
	day 0–21	−1.65% (−2.63 to −0.66)	0.52 (0.35 to 0.79)	0.002
	day 22–365	−1.55% (−2.82 to −0.29)	0.71 (0.52 to 0.97)	0.032
Cerebrovascular disorders				
	day 0–21	−0.69% (−1.26 to −0.11)	0.51 (0.28 to 0.95)	0.034
	day 22–365	−0.38% (−1.04 to 0.28)	0.70 (0.43 to 1.14)	0.154
Dysrhythmia				
	day 0–21	−0.86% (−1.57 to −0.15)	0.57 (0.36 to 0.91)	0.019
	day 22–365	−1.24% (−2.21 to −0.28)	0.58 (0.39 to 0.87)	0.008
Ischemic heart disease				
	day 0–21	−0.85% (−1.39 to −0.31)	0.44 (0.26 to 0.75)	0.003
	day 22–365	−0.01% (−0.73 to 0.70)	1.05 (0.62 to 1.80)	0.845
Inflammatory heart disease				
	day 0–21	0.17% (−0.04 to 0.38)	15.73 (0.98 to 251.39)	0.051
	day 22–365	NA	NA	NA
Other cardiac disorders				
	day 0–21	−0.64% (−1.30 to 0.01)	0.44 (0.21 to 0.92)	0.030
	day 22–365	−0.49% (−1.59 to 0.61)	0.74 (0.49 to 1.11)	0.146
Thrombotic disorders				
	day 0–21	−0.05% (−0.27 to 0.16)	0.62 (0.15 to 2.58)	0.509
	day 22–365	0.01% (−0.40 to 0.43)	0.97 (0.38 to 2.48)	0.947

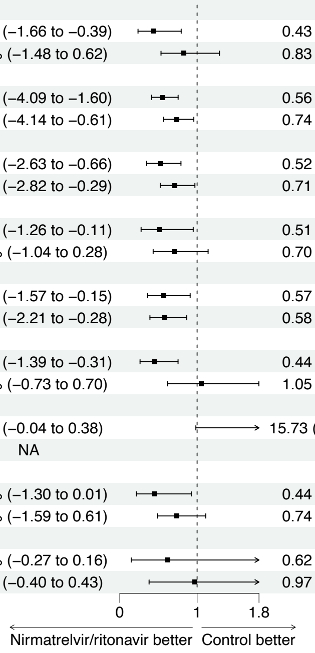

(B) Molnupiravir trial

Outcome	Outcome assessment period	Adjusted RD (95% CI)	Adjusted HR (95% CI)	P value
Cardiovascular mortality				
	day 0–21	−1.32% (−1.98 to −0.65)	0.59 (0.41 to 0.85)	0.004
	day 22–365	−0.19% (−1.24 to 0.86)	0.93 (0.69 to 1.24)	0.607
Composite cardiovascular complications				
	day 0–21	−1.87% (−3.09 to −0.65)	0.67 (0.53 to 0.84)	<0.001
	day 22–365	−0.66% (−2.33 to 1.00)	0.88 (0.73 to 1.08)	0.218
MACE				
	day 0–21	−1.65% (−2.62 to −0.68)	0.62 (0.47 to 0.82)	0.001
	day 22–365	0.23% (−1.14 to 1.61)	1.00 (0.79 to 1.27)	0.982
Cerebrovascular disorders				
	day 0–21	−0.77% (−1.26 to −0.28)	0.57 (0.34 to 0.95)	0.032
	day 22–365	−0.40% (−1.18 to 0.39)	0.81 (0.53 to 1.24)	0.339
Dysrhythmia				
	day 0–21	−0.21% (−0.98 to 0.55)	0.83 (0.58 to 1.19)	0.310
	day 22–365	−0.82% (−1.75 to 0.12)	0.75 (0.54 to 1.05)	0.090
Ischemic heart disease				
	day 0–21	−0.21% (−0.93 to 0.50)	0.75 (0.52 to 1.08)	0.120
	day 22–365	0.56% (−0.25 to 1.37)	1.17 (0.81 to 1.68)	0.405
Inflammatory heart disease				
	day 0–21	0.06% (−0.07 to 0.18)	3.62 (0.22 to 59.38)	0.368
	day 22–365	0.08% (−0.15 to 0.31)	1.96 (0.48 to 8.10)	0.351
Other cardiac disorders				
	day 0–21	−1.01% (−1.65 to −0.36)	0.65 (0.43 to 0.98)	0.040
	day 22–365	−0.07% (−1.21 to 1.07)	0.96 (0.72 to 1.28)	0.787
Thrombotic disorders				
	day 0–21	0.14% (−0.14 to 0.43)	1.76 (0.72 to 4.31)	0.216
	day 22–365	0.07% (−0.34 to 0.48)	0.99 (0.52 to 1.89)	0.985

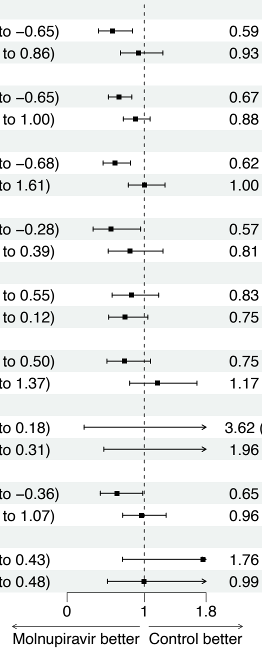

Supplementary Fig. 7. Risk of cardiovascular complications in target trials among unvaccinated patients hospitalized for COVID-19. (A) Target trial of nirmatrelvir/ritonavir (n=2,183) versus no treatment (n=5,427). (B) Target trial of molnupiravir (n=2,107) versus no treatment (6,445). Adjusted HRs (square dots) and 95% CIs (error bars) are presented in (A) and (B). The dashed vertical line in (A) and (B) represents the HR of 1.00. Statistical analysis with two-sided Wald test in (A) and (B).

MACE: major adverse cardiovascular events. RD: risk difference. HR: hazard ratio. CI: confidence interval. NA: Not available due to zero observation.

(A) Nirmatrelvir/ritonavir trial

Outcome	Outcome assessment period	Adjusted RD (95% CI)	Adjusted HR (95% CI)	P value
Cardiovascular mortality				
	day 0–21	−0.89% (−1.20 to −0.58)	0.42 (0.27 to 0.64)	<0.001
	day 22–365	−0.85% (−1.24 to −0.45)	0.52 (0.38 to 0.72)	<0.001
Composite cardiovascular complications				
	day 0–21	−3.03% (−3.73 to −2.32)	0.53 (0.45 to 0.63)	<0.001
	day 22–365	−1.22% (−1.95 to −0.49)	0.75 (0.62 to 0.91)	0.004
MACE				
	day 0–21	−2.10% (−2.61 to −1.59)	0.45 (0.36 to 0.56)	<0.001
	day 22–365	−1.25% (−1.90 to −0.60)	0.71 (0.54 to 0.93)	0.012
Cerebrovascular disorders				
	day 0–21	−0.81% (−1.21 to −0.41)	0.39 (0.27 to 0.55)	<0.001
	day 22–365	−0.48% (−0.89 to −0.07)	0.71 (0.51 to 0.99)	0.041
Dysrhythmia				
	day 0–21	−0.62% (−1.03 to −0.20)	0.74 (0.60 to 0.93)	0.009
	day 22–365	−0.55% (−0.96 to −0.13)	0.69 (0.54 to 0.87)	0.002
Ischemic heart disease				
	day 0–21	−1.21% (−1.57 to −0.85)	0.35 (0.24 to 0.51)	<0.001
	day 22–365	−0.29% (−0.59 to 0.02)	0.67 (0.52 to 0.87)	0.002
Inflammatory heart disease				
	day 0–21	−0.01% (−0.13 to 0.10)	0.58 (0.12 to 2.84)	0.499
	day 22–365	−0.05% (−0.12 to 0.02)	0.63 (0.27 to 1.46)	0.277
Other cardiac disorders				
	day 0–21	−0.79% (−1.06 to −0.53)	0.47 (0.32 to 0.69)	<0.001
	day 22–365	−0.97% (−1.48 to −0.46)	0.65 (0.43 to 0.97)	0.036
Thrombotic disorders				
	day 0–21	−0.11% (−0.23 to 0.01)	1.10 (0.60 to 2.00)	0.762
	day 22–365	−0.16% (−0.34 to 0.02)	0.61 (0.40 to 0.92)	0.018

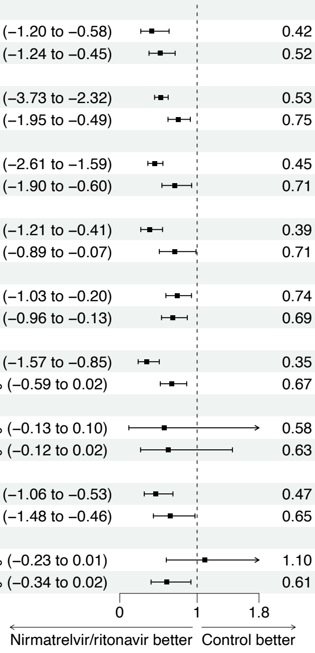

(B) Molnupiravir trial

Outcome	Outcome assessment period	Adjusted RD (95% CI)	Adjusted HR (95% CI)	P value
Cardiovascular mortality				
	day 0–21	−0.99% (−1.37 to −0.62)	0.63 (0.49 to 0.82)	<0.001
	day 22–365	−0.43% (−0.86 to 0.00)	0.75 (0.60 to 0.92)	0.007
Composite cardiovascular complications				
	day 0–21	−0.96% (−1.77 to −0.16)	0.86 (0.76 to 0.98)	0.023
	day 22–365	−0.26% (−1.04 to 0.52)	0.91 (0.80 to 1.03)	0.145
MACE				
	day 0–21	−0.71% (−1.30 to −0.11)	0.82 (0.69 to 0.97)	0.017
	day 22–365	−0.42% (−1.09 to 0.25)	0.87 (0.74 to 1.01)	0.076
Cerebrovascular disorders				
	day 0–21	−0.69% (−1.10 to −0.29)	0.56 (0.41 to 0.76)	<0.001
	day 22–365	−0.12% (−0.55 to 0.31)	0.90 (0.69 to 1.16)	0.404
Dysrhythmia				
	day 0–21	−0.07% (−0.55 to 0.41)	0.95 (0.78 to 1.15)	0.576
	day 22–365	0.09% (−0.39 to 0.56)	1.00 (0.82 to 1.23)	0.986
Ischemic heart disease				
	day 0–21	0.12% (−0.32 to 0.56)	1.06 (0.84 to 1.34)	0.608
	day 22–365	−0.08% (−0.46 to 0.30)	0.90 (0.71 to 1.15)	0.410
Inflammatory heart disease				
	day 0–21	0.01% (−0.14 to 0.15)	1.25 (0.26 to 6.10)	0.779
	day 22–365	−0.03% (−0.11 to 0.05)	0.70 (0.29 to 1.73)	0.446
Other cardiac disorders				
	day 0–21	−0.58% (−0.94 to −0.21)	0.70 (0.54 to 0.92)	0.011
	day 22–365	−0.24% (−0.76 to 0.29)	0.88 (0.73 to 1.06)	0.181
Thrombotic disorders				
	day 0–21	−0.27% (−0.41 to −0.13)	0.28 (0.13 to 0.61)	0.001
	day 22–365	−0.15% (−0.37 to 0.07)	0.72 (0.47 to 1.10)	0.129

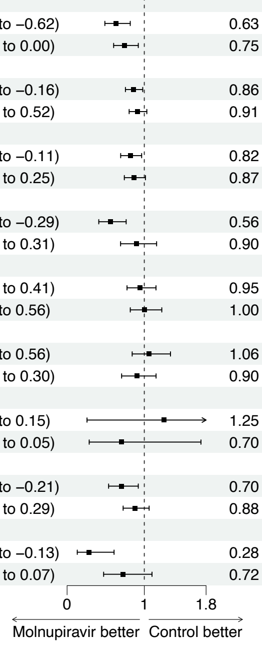

Supplementary Fig. 8. Risk of cardiovascular complications in target trials among vaccinated patients hospitalized for COVID-19. (A) Target trial of nirmatrelvir/ritonavir (n=12,659) versus no treatment (n=14,233). (B) Target trial of molnupiravir (n=7,946) versus no treatment (n=15,718). Adjusted HRs (square dots) and 95% CIs (error bars) are presented in (A) and (B). The dashed vertical line in (A) and (B) represents the HR of 1.00. Statistical analysis with two-sided Wald test in (A) and (B).

MACE: major adverse cardiovascular events. RD: risk difference. HR: hazard ratio. CI: confidence interval.

(A) Nirmatrelvir/ritonavir trial

Outcome	Outcome assessment period	Adjusted RD (95% CI)	Adjusted HR (95% CI)	P value
Cardiovascular mortality				
	day 0–30	−1.08% (−1.37 to −0.80)	0.45 (0.29 to 0.69)	<0.001
	day 31–365	−0.69% (−1.05 to −0.32)	0.64 (0.48 to 0.85)	0.002
Composite cardiovascular complications				
	day 0–30	−3.33% (−3.97 to −2.69)	0.56 (0.48 to 0.65)	<0.001
	day 31–365	−1.29% (−1.91 to −0.68)	0.73 (0.64 to 0.83)	<0.001
MACE				
	day 0–30	−2.33% (−2.81 to −1.85)	0.51 (0.41 to 0.62)	<0.001
	day 31–365	−1.18% (−1.69 to −0.66)	0.67 (0.57 to 0.79)	<0.001
Cerebrovascular disorders				
	day 0–30	−1.13% (−1.49 to −0.77)	0.41 (0.30 to 0.56)	<0.001
	day 31–365	−0.36% (−0.71 to 0.00)	0.74 (0.56 to 0.97)	0.028
Dysrhythmia				
	day 0–30	−0.82% (−1.20 to −0.44)	0.66 (0.55 to 0.80)	<0.001
	day 31–365	−0.62% (−0.97 to −0.26)	0.65 (0.53 to 0.80)	<0.001
Ischemic heart disease				
	day 0–30	−1.17% (−1.49 to −0.85)	0.41 (0.31 to 0.55)	<0.001
	day 31–365	−0.19% (−0.47 to 0.09)	0.77 (0.60 to 0.98)	0.035
Inflammatory heart disease				
	day 0–30	−0.01% (−0.06 to 0.04)	1.21 (0.28 to 5.16)	0.795
	day 31–365	−0.06% (−0.12 to 0.00)	0.47 (0.20 to 1.11)	0.084
Other cardiac disorders				
	day 0–30	−0.89% (−1.15 to −0.64)	0.59 (0.41 to 0.86)	0.005
	day 31–365	−0.96% (−1.38 to −0.54)	0.61 (0.48 to 0.76)	<0.001
Thrombotic disorders				
	day 0–30	−0.14% (−0.27 to −0.01)	1.05 (0.49 to 2.25)	0.898
	day 31–365	−0.07% (−0.24 to 0.10)	0.76 (0.49 to 1.17)	0.208

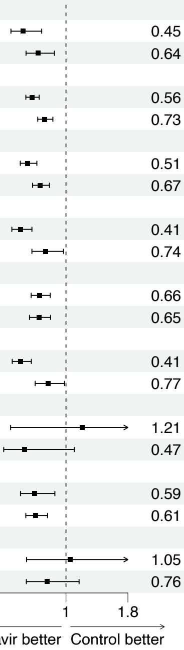

(B) Molnupiravir trial

Outcome	Outcome assessment period	Adjusted RD (95% CI)	Adjusted HR (95% CI)	P value
Cardiovascular mortality				
	day 0–30	−1.16% (−1.52 to −0.80)	0.65 (0.53 to 0.80)	<0.001
	day 31–365	−0.20% (−0.59 to 0.19)	0.86 (0.72 to 1.04)	0.122
Composite cardiovascular complications				
	day 0–30	−1.62% (−2.32 to −0.93)	0.78 (0.70 to 0.87)	<0.001
	day 31–365	0.04% (−0.64 to 0.73)	0.96 (0.85 to 1.07)	0.439
MACE				
	day 0–30	−1.23% (−1.75 to −0.70)	0.73 (0.64 to 0.84)	<0.001
	day 31–365	−0.03% (−0.62 to 0.56)	0.95 (0.82 to 1.09)	0.473
Cerebrovascular disorders				
	day 0–30	−0.92% (−1.25 to −0.58)	0.55 (0.42 to 0.71)	<0.001
	day 31–365	−0.06% (−0.41 to 0.30)	0.94 (0.75 to 1.18)	0.598
Dysrhythmia				
	day 0–30	−0.27% (−0.70 to 0.16)	0.87 (0.74 to 1.02)	0.087
	day 31–365	0.04% (−0.37 to 0.44)	0.99 (0.82 to 1.19)	0.902
Ischemic heart disease				
	day 0–30	−0.09% (−0.46 to 0.28)	0.92 (0.77 to 1.11)	0.405
	day 31–365	0.09% (−0.24 to 0.42)	0.99 (0.80 to 1.24)	0.951
Inflammatory heart disease				
	day 0–30	−0.05% (−0.12 to 0.02)	0.85 (0.24 to 3.00)	0.803
	day 31–365	0.03% (−0.06 to 0.12)	1.16 (0.49 to 2.74)	0.740
Other cardiac disorders				
	day 0–30	−0.71% (−1.05 to −0.37)	0.71 (0.58 to 0.88)	0.002
	day 31–365	−0.04% (−0.52 to 0.43)	0.94 (0.79 to 1.12)	0.498
Thrombotic disorders				
	day 0–30	−0.17% (−0.33 to −0.01)	0.58 (0.36 to 0.93)	0.024
	day 31–365	−0.07% (−0.25 to 0.10)	0.80 (0.54 to 1.19)	0.277

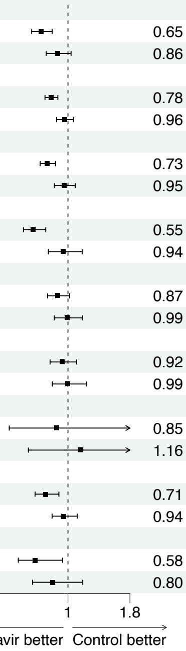

Supplementary Fig. 9. Risk of cardiovascular complications in target trials of COVID-19 hospitalizations with the outcome ascertainment periods as from 0-30 days or from 31-365 days after the index date. (A) Target trial of nirmatrelvir/ritonavir (n=14,842) versus no treatment (n=19,660). (B) Target trial of molnupiravir (n=10,053) versus no treatment (n=22,163). Adjusted HRs (square dots) and 95% CIs (error bars) are presented in (A) and (B). The dashed vertical line in (A) and (B) represents the HR of 1.00. Statistical analysis with two-sided Wald test in (A) and (B).

MACE: major adverse cardiovascular events. RD: risk difference. HR: hazard ratio. CI: confidence interval.

(A) Nirmatrelvir/ritonavir trial

Outcome	Outcome assessment period	Adjusted RD (95% CI)	Adjusted HR (95% CI)	P value
Cardiovascular mortality				
	day 0–21	−0.96% (−1.24 to −0.68)	0.47 (0.30 to 0.73)	0.001
	day 22–365	−0.65% (−1.03 to −0.27)	0.63 (0.47 to 0.85)	0.003
Composite cardiovascular complications				
	day 0–21	−3.65% (−4.33 to −2.97)	0.55 (0.47 to 0.64)	<0.001
	day 22–365	−1.49% (−2.15 to −0.83)	0.71 (0.62 to 0.81)	<0.001
MACE				
	day 0–21	−2.32% (−2.82 to −1.82)	0.51 (0.42 to 0.63)	<0.001
	day 22–365	−1.11% (−1.64 to −0.58)	0.68 (0.58 to 0.81)	<0.001
Cerebrovascular disorders				
	day 0–21	−1.15% (−1.52 to −0.78)	0.41 (0.30 to 0.57)	<0.001
	day 22–365	−0.36% (−0.73 to 0.01)	0.74 (0.56 to 0.99)	0.042
Dysrhythmia				
	day 0–21	−0.86% (−1.25 to −0.47)	0.64 (0.53 to 0.78)	<0.001
	day 22–365	−0.59% (−0.95 to −0.23)	0.66 (0.53 to 0.82)	<0.001
Ischemic heart disease				
	day 0–21	−1.07% (−1.40 to −0.74)	0.43 (0.32 to 0.58)	<0.001
	day 22–365	−0.16% (−0.44 to 0.13)	0.78 (0.60 to 1.00)	0.054
Inflammatory heart disease				
	day 0–21	−0.02% (−0.08 to 0.04)	0.93 (0.19 to 4.50)	0.925
	day 22–365	−0.06% (−0.12 to 0.01)	0.48 (0.20 to 1.15)	0.098
Other cardiac disorders				
	day 0–21	−0.85% (−1.11 to −0.59)	0.61 (0.42 to 0.89)	0.011
	day 22–365	−0.91% (−1.32 to −0.49)	0.60 (0.47 to 0.76)	<0.001
Thrombotic disorders				
	day 0–21	−0.15% (−0.29 to −0.02)	1.04 (0.47 to 2.27)	0.928
	day 22–365	−0.12% (−0.29 to 0.06)	0.67 (0.42 to 1.05)	0.083

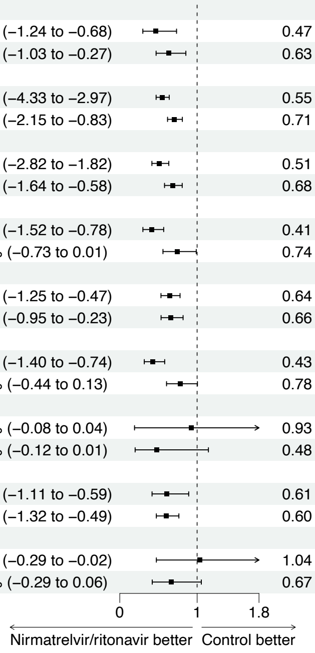

(B) Molnupiravir trial

Outcome	Outcome assessment period	Adjusted RD (95% CI)	Adjusted HR (95% CI)	P value
Cardiovascular mortality				
	day 0–21	−0.99% (−1.38 to −0.61)	0.69 (0.55 to 0.86)	0.001
	day 22–365	−0.18% (−0.58 to 0.22)	0.85 (0.70 to 1.04)	0.110
Composite cardiovascular complications				
	day 0–21	−1.46% (−2.24 to −0.68)	0.81 (0.73 to 0.90)	<0.001
	day 22–365	0.20% (−0.55 to 0.94)	0.98 (0.88 to 1.10)	0.758
MACE				
	day 0–21	−1.02% (−1.60 to −0.44)	0.78 (0.68 to 0.89)	<0.001
	day 22–365	0.03% (−0.58 to 0.64)	0.97 (0.84 to 1.11)	0.630
Cerebrovascular disorders				
	day 0–21	−0.90% (−1.29 to −0.50)	0.55 (0.41 to 0.72)	<0.001
	day 22–365	−0.10% (−0.48 to 0.27)	0.94 (0.74 to 1.19)	0.618
Dysrhythmia				
	day 0–21	−0.22% (−0.68 to 0.23)	0.88 (0.74 to 1.04)	0.145
	day 22–365	−0.01% (−0.43 to 0.41)	0.97 (0.80 to 1.17)	0.734
Ischemic heart disease				
	day 0–21	0.05% (−0.35 to 0.45)	0.99 (0.81 to 1.20)	0.905
	day 22–365	0.04% (−0.30 to 0.39)	0.98 (0.78 to 1.23)	0.849
Inflammatory heart disease				
	day 0–21	−0.01% (−0.12 to 0.11)	1.01 (0.25 to 4.05)	0.986
	day 22–365	0.03% (−0.06 to 0.12)	1.21 (0.49 to 3.00)	0.678
Other cardiac disorders				
	day 0–21	−0.59% (−0.96 to −0.22)	0.77 (0.61 to 0.97)	0.028
	day 22–365	−0.04% (−0.52 to 0.43)	0.95 (0.79 to 1.13)	0.539
Thrombotic disorders				
	day 0–21	−0.17% (−0.35 to 0.01)	0.62 (0.38 to 1.01)	0.055
	day 22–365	−0.11% (−0.28 to 0.06)	0.70 (0.47 to 1.07)	0.099

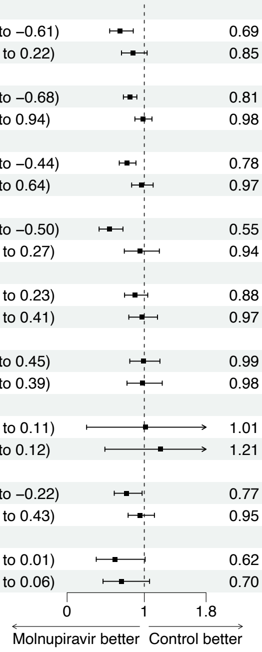

Supplementary Fig. 10. Risk of cardiovascular complications in target trials of COVID-19 hospitalizations without a history of related outcomes within 6 years before the index date. (A) Target trial of nirmatrelvir/ritonavir (n=14,180) versus no treatment (n=18,620). (B) Target trial of molnupiravir (n=9,060) versus no treatment (n=20,895). Adjusted HRs (square dots) and 95% CIs (error bars) are presented in (A) and (B). The dashed vertical line in (A) and (B) represents the HR of 1.00. Statistical analysis with two-sided Wald test in (A) and (B).

MACE: major adverse cardiovascular events. RD: risk difference. HR: hazard ratio. CI: confidence interval.

(A) Nirmatrelvir/ritonavir trial

Outcome	Outcome assessment period	Adjusted RD (95% CI)	Adjusted subhazard ratio (95% CI)	P value
Cardiovascular mortality				
	day 0–21	−0.97% (−1.24 to −0.71)	0.47 (0.30 to 0.76)	0.002
	day 22–365	−0.81% (−1.19 to −0.43)	0.63 (0.48 to 0.81)	<0.001
Composite cardiovascular complications				
	day 0–21	−3.02% (−3.64 to −2.41)	0.56 (0.47 to 0.67)	<0.001
	day 22–365	−1.50% (−2.15 to −0.85)	0.76 (0.65 to 0.88)	<0.001
MACE				
	day 0–21	−2.13% (−2.58 to −1.67)	0.49 (0.40 to 0.60)	<0.001
	day 22–365	−1.23% (−1.80 to −0.67)	0.72 (0.58 to 0.89)	0.003
Cerebrovascular disorders				
	day 0–21	−0.91% (−1.26 to −0.57)	0.45 (0.32 to 0.62)	<0.001
	day 22–365	−0.53% (−0.89 to −0.16)	0.68 (0.53 to 0.88)	0.004
Dysrhythmia				
	day 0–21	−0.67% (−1.03 to −0.31)	0.71 (0.58 to 0.86)	<0.001
	day 22–365	−0.73% (−1.10 to −0.35)	0.65 (0.54 to 0.79)	<0.001
Ischemic heart disease				
	day 0–21	−1.14% (−1.45 to −0.83)	0.40 (0.29 to 0.56)	<0.001
	day 22–365	−0.21% (−0.49 to 0.08)	0.78 (0.62 to 0.98)	0.036
Inflammatory heart disease				
	day 0–21	−0.02% (−0.07 to 0.04)	1.31 (0.27 to 6.32)	0.739
	day 22–365	−0.06% (−0.12 to 0.00)	0.48 (0.22 to 1.09)	0.079
Other cardiac disorders				
	day 0–21	−0.87% (−1.12 to −0.63)	0.53 (0.33 to 0.86)	0.010
	day 22–365	−0.84% (−1.28 to −0.39)	0.72 (0.53 to 0.98)	0.039
Thrombotic disorders				
	day 0–21	−0.11% (−0.22 to 0.01)	1.31 (0.58 to 2.95)	0.519
	day 22–365	−0.10% (−0.28 to 0.08)	0.72 (0.48 to 1.07)	0.105

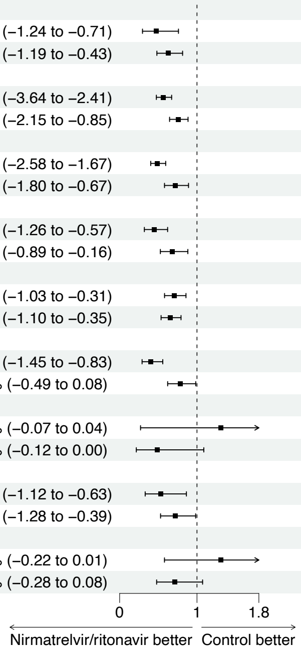

(B) Molnupiravir trial

Outcome	Outcome assessment period	Adjusted RD (95% CI)	Adjusted subhazard ratio (95% CI)	P value
Cardiovascular mortality				
	day 0–21	−1.03% (−1.37 to −0.69)	0.68 (0.54 to 0.84)	<0.001
	day 22–365	−0.37% (−0.78 to 0.03)	0.82 (0.68 to 0.97)	0.023
Composite cardiovascular complications				
	day 0–21	−1.36% (−2.03 to −0.70)	0.82 (0.74 to 0.92)	0.001
	day 22–365	−0.29% (−1.00 to 0.43)	0.92 (0.83 to 1.03)	0.136
MACE				
	day 0–21	−1.02% (−1.52 to −0.53)	0.77 (0.67 to 0.89)	<0.001
	day 22–365	−0.24% (−0.85 to 0.38)	0.92 (0.81 to 1.05)	0.238
Cerebrovascular disorders				
	day 0–21	−0.74% (−1.06 to −0.43)	0.59 (0.45 to 0.78)	<0.001
	day 22–365	−0.18% (−0.55 to 0.20)	0.89 (0.71 to 1.10)	0.277
Dysrhythmia				
	day 0–21	−0.14% (−0.55 to 0.26)	0.94 (0.79 to 1.11)	0.457
	day 22–365	−0.10% (−0.53 to 0.32)	0.93 (0.79 to 1.11)	0.420
Ischemic heart disease				
	day 0–21	−0.04% (−0.40 to 0.32)	0.98 (0.80 to 1.19)	0.818
	day 22–365	0.03% (−0.31 to 0.38)	0.97 (0.79 to 1.19)	0.751
Inflammatory heart disease				
	day 0–21	−0.04% (−0.10 to 0.03)	1.01 (0.27 to 3.83)	0.984
	day 22–365	0.01% (−0.08 to 0.10)	1.05 (0.46 to 2.39)	0.900
Other cardiac disorders				
	day 0–21	−0.66% (−0.99 to −0.34)	0.75 (0.60 to 0.94)	0.013
	day 22–365	−0.12% (−0.62 to 0.37)	0.93 (0.79 to 1.09)	0.376
Thrombotic disorders				
	day 0–21	−0.16% (−0.29 to −0.02)	0.56 (0.32 to 1.00)	0.048
	day 22–365	−0.10% (−0.30 to 0.10)	0.80 (0.56 to 1.15)	0.229

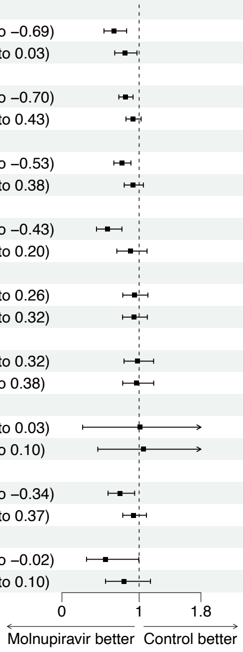

Supplementary Fig. 11. Risk of cardiovascular complications in target trials of COVID-19 hospitalizations using the Fine-Gray model. (A) Target trial of nirmatrelvir/ritonavir (n=14,842) versus no treatment (n=19,660). (B) Target trial of molnupiravir (n=10,053) versus no treatment (n=22,163). Adjusted HRs (square dots) and 95% CIs (error bars) are presented in (A) and (B). The dashed vertical line in (A) and (B) represents the HR of 1.00. Statistical analysis with two-sided Wald test in (A) and (B).
MACE: major adverse cardiovascular events. RD: risk difference. CI: confidence interval.
